# A facile approach for the synthesis of porous hematite and magnetite nanoparticles through sol-gel self-combustion

**DOI:** 10.3906/kim-2104-59

**Published:** 2021-09-12

**Authors:** Imene GRITLI, Afrah BARDAOUI, Jamila BEN NACEUR, Salah AMMAR, Mohammad ABU HAIJA, Sherif Mohamed Abdel Salam KESHK, Radhouane CHTOUROU

**Affiliations:** 1 Nanomaterials and Systems for Renewable Energy Laboratory, Research and Technology Center of Energy, Technopark Borj Cedria, Hammam Lif, Tunisia; 2 Faculty of Sciences of Tunisia, University of Tunisia El Manar, El Manar, Tunisia; 3 Department of Chemistry, Khalifa University of Science and Technology, Abu Dhabi, United Arab Emirates

**Keywords:** Fe_3_O_4_ nanoparticles, hematite, starch, sol-gel auto combustion

## Abstract

Porous magnetite (Fe_3_O_4_) and hematite (α-Fe_2_O_3_) nanoparticles were prepared via the sol-gel auto-combustion method. The gels were prepared by reacting ferric nitrates (as oxidants) with starch (as fuel) at an elevated temperature of 200 °C. Different ratios (Φ) of ferric nitrates to starch were used for the synthesis (Φ = fuel/oxidant). The synthesized iron oxides were characterized by Fourier transform infrared (FT-IR) spectroscopy, Raman spectroscopy, X-ray diffraction (XRD) spectroscopy, scanning electron microscopy (SEM), transmission electron microscopy (TEM), Brunauer–Emmet–Teller (BET) and vibrating sample magnetometer (VSM) analysis techniques. The crystal structure, morphology, and specific surface area of the iron oxide nanoparticles (Fe_3_O_4_ and α-Fe_2_O_3_) were found to be dependent on the starch content. The FT-IR, XRD and VSM analysis of the iron oxides for Φ = 0.3 and 0.7 confirmed the formation of the α-Fe_2_O_3_ core, whereas at Φ = 1, 1.7, and 2 showed that Fe_3_O_4_ cores were formed with the highest saturation magnetization of 60.36 emu/g at Φ = 1. The morphology of the Fe_3_O_4_ nanoparticles exhibited a quasi-spherical shape, while α-Fe_2_O_3_ nanoparticles appeared polygonal and formed clusters. The highest specific surface area was found to be 48 m^2^ g^−^^1^ for Φ = 1.7 owing to the rapid thermal decomposition process. Type II and type III isotherms indicated mesoporous structures.

## 1. Introduction

Nanoparticles (NPs) are at the front of quick advancement in nanotechnology. These indispensable and superior materials exhibiting elite size-subordinate properties can find their applications in various fields [1]. In general, iron oxide NPs are widespread and commonly used. They can find their applications in many biological and industrial activities [2,3]. The three most widespread forms of iron oxides in nature are magnetite (Fe_3_O_4_), maghemite (Φ-Fe_2_O_3_), and hematite (α-Fe_2_O_3_). Magnetic Fe_3_O_4_ and α-Fe_2_O_3_ NPs can be potentially used in the field of biomedicine as they exhibit low toxicity and superparamagnetic properties [4,5]. Fe_3_O_4_ exhibits a cubic inverse spinel structure and can be utilized for developing storage media and ferrofluids. They can be used for treating hyperthermia [6,7] and in the fields of drug delivery [8], biomedicine [9,10] and catalysis [11,12].

Iron oxide NPs with suitable surface properties can be prepared via several techniques such as physical, biological, and chemical procedures. The physical methods, such as pulsed laser deposition [13], are easily executed but difficult to control the nanometric size of the prepared particles and may require high temperatures [14–16]. The biological methods [17] are of low cost but relatively slow [14,16]. The chemical methods, such as coprecipitation [18–20], sol-gel synthesis [21–23], template-assisted synthesis [24], reverse micelle [25], hydrothermal [26], solvothermal [27], sonochemical [28,29], combustion synthesis [30–34], electrodeposition [35], and pyrolysis [36] are of low cost and high yield products in which size, composition and the shape of nanoparticles can be controlled [14,16]. Among these chemical methods, the coprecipitation is an easy and rapid procedure at low temperatures (<100 °C). However, the coprecipitation procedure has some restrictions as the exploit of nonenvironmental-friendly chemicals and a limit to control size and shape of final product [14,16]. The hydrothermal procedure normally produces NPs with a high crystallinity degree, but it needs higher temperatures than the coprecipitation [16]. Sol-gel self-combustion route is a fast preparation route. The advantages of this route include the use of few precursors and the formation of nontoxic byproducts. A minimum number of preparation steps are involved, and the efficiency of the scale-up process is high. The final products can be obtained in high yields [35]. In the sol-gel method, usually referred to as the sol-gel auto combustion, sol-gel autoignition, or sol-gel self-combustion method. The nitrates of the constituent metal ions (oxidant) and a suitable organic compound (a chelating agent that ignites the reaction; fuel), such as urea, glycine, and citric acid are used as the precursors, owing to their high solubility in water.

The fuel to nitrate ratios can significantly affect the particle size and magnetic properties of the NPs. The equivalence ratio of an oxidant and fuel mixture (Φ) can be computed in terms of the elemental stoichiometric coefficient [37]. The influence of the chelating/combustion/fuel agents on the structural and magnetic properties of iron oxide has been previously reported [38,39]. The fuel/oxidant ratio (Φ) significantly affects the flame temperature and the whole burning cycle. Therefore, the particles morphology, crystalline phase, and surface area of the particles can be controlled by manipulating the combustion temperature. A group of organic compounds (e.g., citric acid, urea, glycine, oxalyl di-hydrazide, carbohydrazide, tetra form triazine, *N*, *N*-diformylhydrazine, and hexamethylene tetra mine) have been used in the combustion process as high-temperature fuels [40]. Recently, the influence of citric acid, Cetyltrimethylammonium bromide (CTAB), urea, and glycine has been determined [30,34], and a single phase of Fe_3_O_4_ exhibiting a high specific surface area was obtained. The synthesis of hematite nanoparticles using microwave assisted sol-gel auto-combustion method using ferric nitrate as oxidizer and a mixture of urea and glycine fuel as reducer [32]. The results showed that the powders were composed of polycrystalline oxides with a crystallite size of 30 nm of gamma Fe_2_O_3_ phase without sintering. In addition, the electrical conductivity of nano Fe_2_O_3_ in pellet form increases with the increase in frequency of impedance analyzer owing to hopping of charge carriers amongst localized sites [32]. On the other hand, starch (α1,4-glucan) is one of the most widely used polysaccharides as it is widely available, biodegradable, safe, and cheap [41]. Its physicochemical properties (for example, hydrophilicity, high chemical reactivity, chirality, chelation, flexible matrix, and adsorption capacities) have helped expand the green arrangements or strong state-based planning of metal oxide NPs [33,42].

Furthermore, some improved kinds of starch produced enzymatically (porous starch) and synthetically (functionalized starch) show the option to coordinate the sizes and states of the metal oxide NPs [39,43]. The facile preparation approach for both hematite and magnetite is essential to facilitate their use in several industrial fields. Herein we report a novel sol-gel auto-combustion synthesis of porous hematite and magnetite nanoparticles in which starch has been used as single-fuel at a low temperature (200 °C) for the first time. The effects of the starch fuel composition ratio on the crystallite sizes, morphologies, and surface areas were evaluated.

## 2. Materials and methods

### 2.1. Chemicals

Ferric nitrate nonahydrate Fe (NO_3_)_3_.9H_2_O (404 g/mol molecular weight; ≥ 98 % purity) and ammonia solution (17.03 g/mol molecular weight; 28 % purity) were purchased from SIGMA-ALDRICH. Commercial starch powder (C_6_H_10_O_5_) _n_, extracted from cereal grains such as corn, was purchased from Tunisia [44].

### 2.2. Synthesis of MNPs

The magnetic iron oxide nanoparticles (MNPs) were prepared by the sol-gel autoignition strategy utilizing Fe (NO_3_)_3_.9H_2_O as the oxidant and starch as the fuel. The relative fuel-to-oxidant ratio (Φ) was determined utilizing the following equation [45]:


(1) $$\Phi =\frac{\text{n}.\text{VR}}{-\text{m}.\text{VO}}$$

where n and m denote the mole fraction of the monomer of starch and ferric nitrate, respectively. VR and VO represent the total reducing and oxidizing valences of the distinctive crude material respectively. C and H atoms are considered as reducing elements with the corresponding valences of +4 and −1, whereas O is considered as an oxidizing element with the valency of −2. N is assumed to have a valency of 0. The valency of Fe is considered as +3. In this work, the reduction valence of the monomer of starch (C_6_H_10_O_5_) is (24 + 10 − 10) = + 24, whereas the valence of the metal nitrate, Fe (NO_3_)_3_ is (3 − 18) = − 15. Here, the relative fuel-to-oxidant ratio is considered fuel-lean when Φ < 1, stoichiometric when Φ = 1, and fuel-rich when Φ > 1. On the base of the propellant chemistry [45], the redox processes occurring during the combustion process can be outlined as follows:

Fuel-lean condition:


$$6\text{Fe }{\left({\text{NO}}_{3}\right)}_{3}+\frac{15\Phi }{4}{\text{C}}_{6}{\text{H}}_{10}{\text{O}}_{5}+\frac{45}{2}(\Phi -1)\;{\text{O}}_{2}\to 3{\text{Fe}}_{2}{\text{O}}_{3}+\frac{45\Phi }{2}{\text{CO}}_{2}+\frac{75\Phi }{4}{\text{H}}_{2}\text{O}+9\;{\text{N}}_{2}$$

Fuel-rich condition:


(3) $$6\text{Fe }{\left({\text{NO}}_{3}\right)}_{3}+\frac{15\Phi }{4}{\text{C}}_{6}{\text{H}}_{10}{\text{O}}_{5}+\left(\frac{45\Phi }{2}-23\right)\;{\text{O}}_{2}\to 2{\text{Fe}}_{3}{\text{O}}_{4}+\frac{45\Phi }{2}{\text{CO}}_{2}+\frac{75\Phi }{4}{\text{H}}_{2}\text{O}+9\;{\text{N}}_{2}$$

Different molar ratios of starch (variable) to Fe (NO_3_)_3_.9H_2_O (constant) [(C_6_H_10_O_5_)_n_: Fe(NO_3_)_3_.9H_2_O = 0.2, 0.4, 0.6, 1, and 1.3] were used to study the role of starch to ferric ratio on the innovative combustion process. The ignition temperature was kept constant. The fuel-to-oxidant ratio Φ (Eq. 1) is presented in Table 1 for fuel lean (Φ = 0.3 and 0.7), stoichiometric (Φ = 1), and fuel rich (Φ = 1.7 and 2) conditions. A typical synthesis procedure is: ferric nitrate monohydrate was dissolved in 50 mL of water followed by the addition of a specific amount of starch (Table 1). The components of the mixture were mixed for 30 min to produce a homogeneous solution. Following this, an alkali solution was added dropwise to adjust the pH to 7. Maintaining a pH during the synthesis at 7 was important to keep the stability of starch [46]. Thus, a solution with pH 7 insures a better stability of metal starch solution [47]. Subsequently, the obtained solution was mixed until a transparent sol was formed, which was dried at 95 °C over a period of 48 h. Following this, a hydrated iron gel was obtained. Combustion of the product was carried out at a temperature of 200 °C, over 1 h in a muffle furnace. Upon heating, the gel underwent a violent exothermic reaction which propagated spontaneously. This was accompanied by a release of gases. At the end of the combustion reaction, the voluminous and fragile foam was produced. After cooling the mixture to room temperature, the foam was ground using an agate mortar, and the iron oxide nanoparticles at various fuel-to-oxidant proportions (Φ = 0.3, 0.7, 1, 1.7, and 2) were obtained.

### 2.3. Spectroscopy measurements

FT-IR spectra (KBr pellets) were recorded on a VERTEX 80 spectrometer in the range of 400–4000 cm^−1^.

Raman spectra were recorded at room temperature using a Raman HORIBA Jobin-Yvon spectrometer: Lab Ram H and argon laser at 488 nm were used. For each sample, three distinct points were placed and measured between 100 and 800 cm^−1^.

UV-vis spectra with diffuse reflectance spectroscopy (UV-vis DRS) were recorded with PerkinElmer Lambda 950 spectrophotometer in the wavelength range of 400–800 nm at room temperature.

### 2.4. X-ray diffraction (XRD)

Structural characterizations of the oxide samples were performed using the XRD Bruker Model: D8 advance X-ray diffractometer under conditions of CuK*α* radiation (λ= 0.15418 nm). The system was operated at 40 kV and 30 mA. Diffraction patterns were recorded in the 2θ range of 10°–70°. The crystallite size of the obtained powder was calculated from the peak of (311) using the Debye Scherrer formula [48]:


(4) $$\text{D}=\frac{0.9\times \lambda }{\beta \times \text{cos }\theta }$$

where D represents the crystallite size in nm, λ is the radiation wavelength (λ = 0.15406 nm), β denotes the full width at half of the maximum of the diffraction lines in radians, and θ represents the Bragg-angle. In addition, structural phase and crystallite size were determined by the Rietveld refinement analysis, using FullProf program.

### 2.5. Field emission scanning electron microscopy (FESEM)

The surface morphologies of the synthesized powder were observed using the Philips XL30 SFEG FESEM instrument equipped with an energy-dispersive spectrometer (EDS). The chemical composition of the samples was analyzed.

### 2.6. High-resolution transmission electron microscopy (HRTEM)

The size distribution was evaluated by studying the TEM images recorded using the TECHNAI 20-Philips instrument (G20, 200 kV). The powders were ultrasonically dispersed in ethanol.

### 2.7. Thermogravimetric analysis (TGA)

Thermal decomposition of the dried gel was studied using the thermogravimetric analysis and differential scanning calorimetry (TG/DSC) techniques (TA instrument; model no. 2950 New Castle, DE with a heating rate of 10 °C/min under nitrogen atmosphere).

### 2.8. Brunauer–Emmett–Teller (BET) surface area measurements

The porosity and the specific surface area of the oxides were determined by the BET nitrogen gas adsorption-desorption analysis conducted at 77 K using the Micromeritics ASAP 2020 instrument. The pore size distribution was evaluated using the Barrett–Joyner–Halenda (BJH) method.

### 2.9. Magnetic properties

The magnetic properties of the produced particles were measured by a Quantum Design PPMS magnetometer. Their isothermal 300 K dc-magnetization M was measured by cycling the magnetic field H between +70 and −70 kOe.

## 3. Results and discussion

### 3.1. FTIR analysis

**Figure 1:**
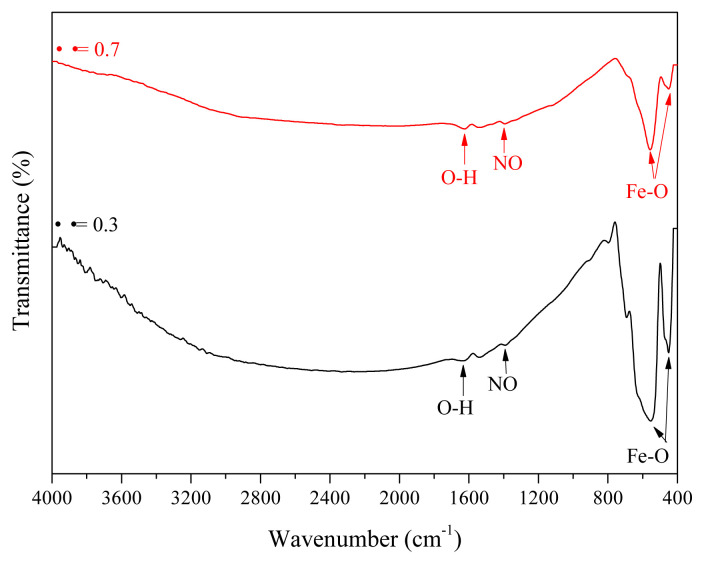
FTIR spectral profile of the resultant iron oxide at different starch to ferric nitrate ratios (Φ = 0.3 and 0.7).

**Figure 2:**
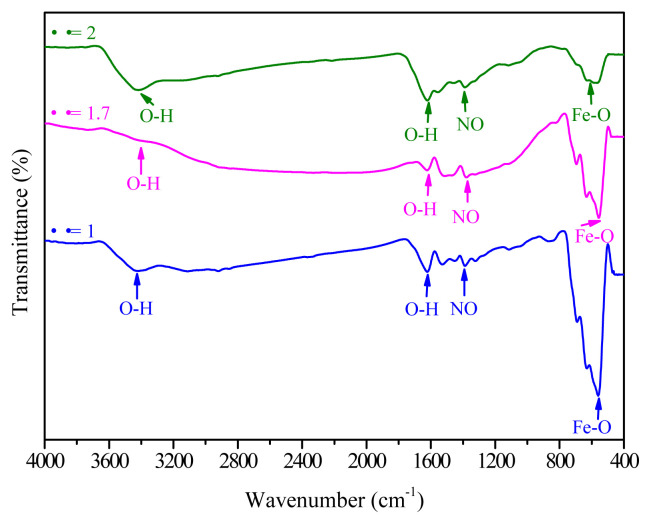
FTIR spectral profile of the resultant iron oxide at different starch to ferric nitrate ratios (Φ = 1, 1.7, and 2).

FTIR spectral profile recorded for the prepared iron oxide NPs under conditions of Φ = 0.3 and 0.7 after the calcination process at 200 °C (Figure 1) showed the absence of bands corresponding to the aliphatic groups derived from starch fuel. In the range of 800 to 400 cm^−1^, the Fe-O vibrational bands corresponding to hematite at 450 cm^−1^ and 567 cm^−1^ were recorded (Figure 1). The bands at 567 cm^−1^ and 450 cm^−1^ can be attributed to the transverse absorption (Eu) of α-Fe_2_O_3_ structure [49]. This result confirms the formation of Fe_2_O_3_ under conditions of low fuel composition ratio. Figure 2 reveals the characteristic bands at 1632 cm^−1^ corresponding to the stretching and bending vibrations of OH adsorbed on the surface of the α-Fe_2_O_3_ under conditions of low calcination temperature [50]. On the other hand, the spectrum displayed the presence of a small band at 1383 cm^−1^ that can be attributed to the NO stretching band originating from the residual ferric nitrate precursor [50,51]. Figure 2 exhibits a strong absorption vibrational band attributable to Fe-O (in Fe_3_O_4_) at 567 cm^−1^ when Φ = 1, 1.7, and 2 [20]. Furthermore, Figure 2 indicates the presence of the stretching and bending OH vibrations (originating from water; adsorbed on the surface of the formed Fe_3_O_4_) at approximately 3439 cm^−1^ and 1632 cm^−1^, respectively [51]. Moreover, the FT-IR spectral profile of Fe_3_O_4_ NPs also revealed the presence of a small absorption band at 1381 cm^−1^. This peak could be assigned to the NO stretching band originating the residual ferric nitrate precursor [49]. Bands corresponding to the hematite structure were not observed in the spectral profile presented in Figure 2. Therefore, the combustion synthesis process of α-Fe_2_O_3_ and Fe_3_O_4_, using starch as the fuel, is mostly influenced by the nature of the fuel used and the fuel-to-oxidant proportion (Φ).

### 3.2. Analysis of Raman spectra

**Figure 3:**
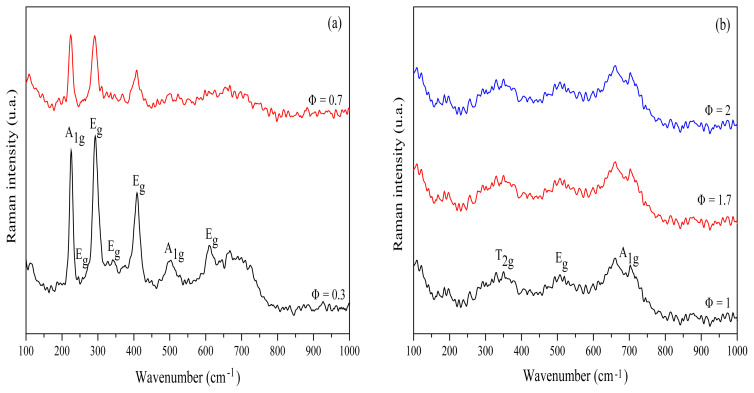
Raman spectral profiles of the resultant iron oxides at different starch to ferric nitrate ratios Φ = 0.3 and 0.7 (a); Φ = 1, 1.7, and 2 (b).

Raman spectra of the resultant iron oxides at different Φ (0.3, 0.7, 1, 1.7, and 2) were recorded using excitation lasers at 488 nm (Figure 3). Generally, hematite belongs to the R-3c crystal space group. Seven phonon mode lines are anticipated in the Raman spectrum: five E_g_ phonon and two A_1 g_ modes [20]. E_g_ modes at 245, 292, 298, 411, and 611 cm^−1^ and A_1 g_ modes at 225 and 496 cm^−1^, can be observed in the spectral profile recorded under conditions of varying Φ (Φ = 0.3 and 0.7; Figure 3a). These results confirm the existence of α-Fe_2_O_3_ at low Φ. Similar results were obtained by analyzing the FT-IR spectral profiles. Raman spectra recorded for Φ = 1, 1.7, and 2 exhibited bands that correspond to the maghemite Φ-Fe_2_O_3_ (Figure 3b)_._ The three Raman active phonon modes (T_2 g_, E_g_, and A_1 g_) of maghemite appeared at 350 cm^−1^ (T_2 g_), 512 cm^−1^ (E_g_) and 703 cm^−1^ (A_1 g_) (Figure 3b) [52]. This transformation to maghemite for Φ = 1, 1.7, and 2 can be potentially attributed to the oxidation of magnetite into maghemite by the heating effect of the incident laser irradiation.

### 3.3. Analysis of XRD patterns

**Figure 4:**
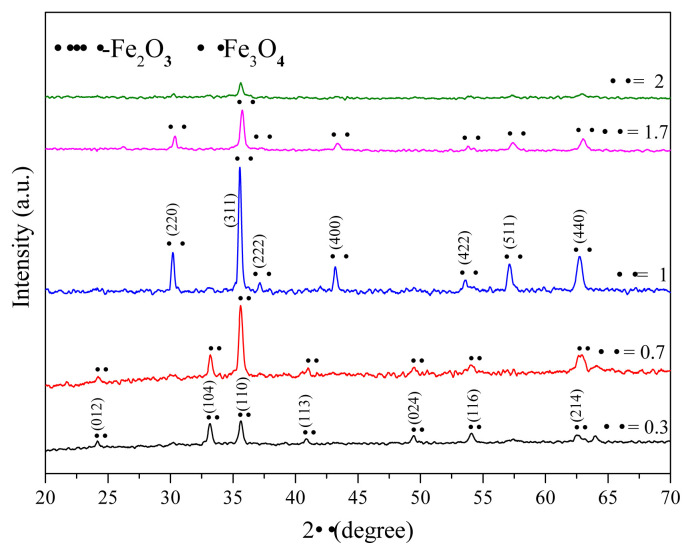
XRD patterns of the resultant oxides at different starch to ferric nitrate ratios (Φ = 0.3, 0.7, 1, 1.7, and 2).

Figure 4 confirmed the presence of the rhombohedral crystallographic phase of α-Fe_2_O_3_ at Φ = 0.3 and 0.7. Seven characteristic peaks for α-Fe_2_O_3_ were allocated at 2θ = 24.19°, 33.12°, 35.67°, 41.0°, 49.50°, 54.0°, and 62.45°). The peaks corresponded to the (012), (104), (110), (113), (024), (116), and (214) planes, respectively (JCPDS cards No.01–086–0550) [53]. The typical XRD pattern of the Fe_3_O_4_ nanoparticles was obtained at Φ = 1, 1.7, and 2 (Figure 4). The XRD patterns recorded with Fe_3_O_4_ revealed the presence of peaks at 30.23° (220), 35.57° (311), 37.29° (222), 43.20° (400), 53.70° (422), 57.24° (511), and 62.86° (440) (JCPDS No.01–075–0033) [31,54]. However, the intensity of the XRD peaks for Fe_3_O_4_ nanoparticles decrease at high Φ value that inhibit the combustion reaction due to the formation of residual carbon on the ion oxide surface [31]. Analysis of the FT-IR, Raman, and XRD spectral profiles revealed that the degree of inversion of the ferric (Fe^3+^) and ferrous (Fe^2+^) ion samples is affected by the fuel composition ratio. At a high starch ratio (Φ ≥ 1), an excessive quantity of gases, such as CO and CO_2_, and heat are produced.

Consequently, the ferric ions (Fe^3+^) get reduced by CO gas to form ferrous ions (Fe ^2+^) according to Equation (5). Subsequently, the magnetic phase is formed.


(5) $$3{\text{Fe}}_{2}{\text{O}}_{3}+\text{CO}\to 2{\text{Fe}}_{3}{\text{O}}_{4}+{\text{CO}}_{2}$$

Furthermore, less amount of energy is required to convert ferric nitrate to hematite than that required to convert it to magnetite.

ΔH for the formation of hematite and magnetite are −823.5 and −1121 KJ/mol, respectively based on previous reports [55].

**Figure 5:**
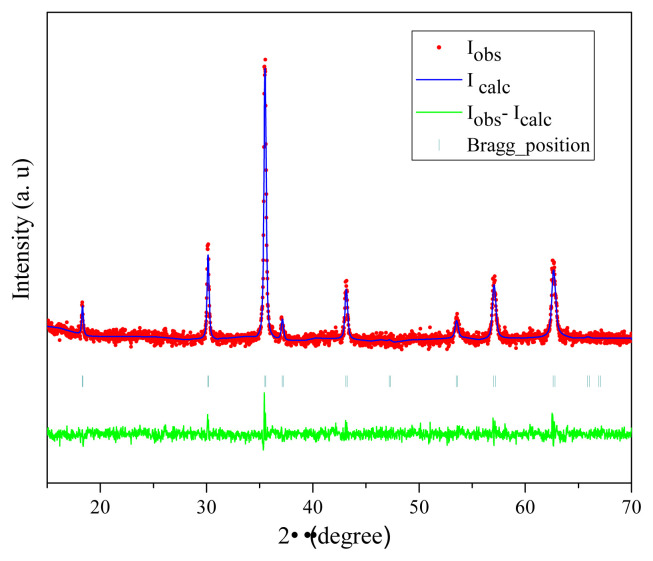
Rietveld refined XRD patterns for synthesized iron oxide nanoparticles at Φ = 1.

The Rietveld refinement results of the synthesized iron oxide nanoparticles at Φ= 1 are illustrated in Figure 5. The experimental spectra are represented by circles while the full line corresponded to the calculated data. The difference between the observed and the calculated pattern are represented in the curve at the bottom. As shown in Figure 5, good agreements can be found between calculated and observable spectra indicating a high fit. The Rietveld parameters goodness of fit (χ^2^), Bragg factor R_B_ and the R_F_ factor were estimated as 1.5; 3.14 and 3.16, respectively. The high agreement between the observed and calculated pattern affirm the formation of Fe_3_O_4_ phase at Φ = 1.

The estimated average crystallite size at Φ = 1 by Rietveld refinement is 37.02 nm while the one calculated using Debye Scherrer formula is 34.71 nm. The average crystallite size from Rietveld analysis is slightly higher than the calculated using Debye Scherrer. This may be due that refinement is determinate using all peaks, but for these calculated from Debye Scherrer formula only (311) peak was considered. The average crystallite obtained by Debye Scherrer formula sizes increased from 26.65 nm at Φ = 0.3 to 32.25 nm at Φ = 0.7. In addition when Φ = 1 the magnetite phase reaches a maximum level (Figure 4) and the crystallite size increased reaching the highest value of 34.71 nm. However, when the fuel-to-oxidant ratio increased to Φ = 1.7 and 2 the crystallite size of Fe_3_O_4_ decreased to 31.27 nm and 25.9 nm, respectively, and fine particles were formed. This can be explained by the presence of the residual carbon owing to combustion of starch in the Fe_3_O_4_ nanoparticles [31].

### 3.4. Analysis of SEM images

**Figure 6:**
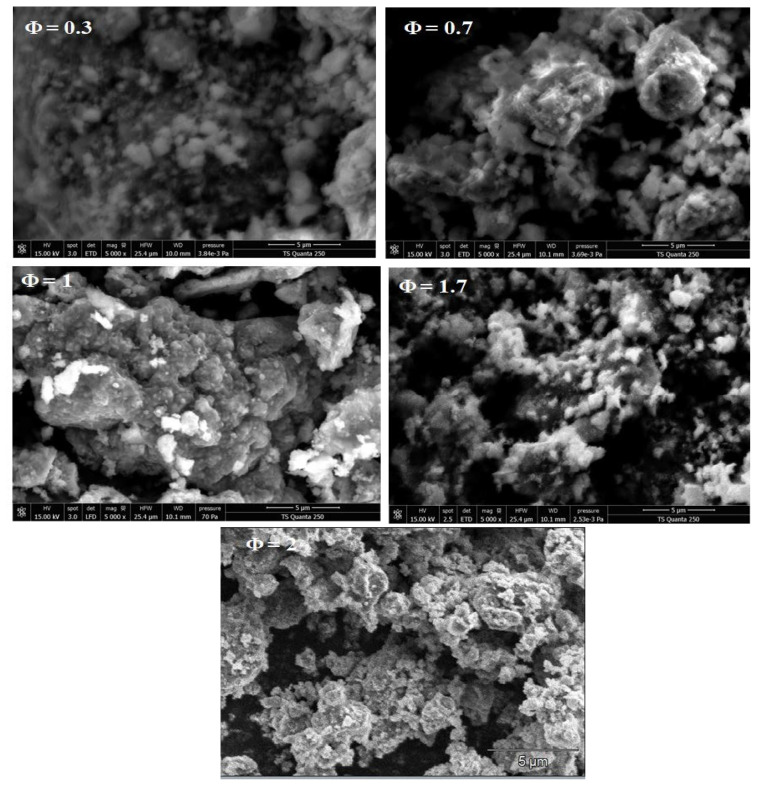
FESEM images of iron oxide nanoparticles at different starch to ferric nitrate ratios (Φ = 0.3, 0.7, 1, 1.7, and 2).

The morphologies of the prepared magnetite nanoparticles at different Φ (1 to 2) were characterized by studying the FESEM images (Figure 6). These magnetite nanoparticles exhibit a quasi-spherical shape that showed excellent rates of internalization. They also exhibited the highest rate of cellular uptake owing to the presence of van der Waals forces among particles that lead to strong agglomeration (Figure 6). The α-Fe_2_O_3_ nanoparticles (at Φ = 0.3 and 0.7) agglomerate with an overall polygonal morphology. For Φ = 0.3 and Φ = 0.7 (fuel-lean condition), the agglomerates imply thick slices.

However, as the amount of fuel is increased in Φ = 1 (stoichiometric equilibrium), the agglomerate forms large clusters. Whereas, for Φ = 1.7 and 2 the densities of the Fe_3_O_4_ nanoparticles decrease as the Fuel ratio increases and the dispersion of the nanoparticles improves [31].

**Figure 7:**
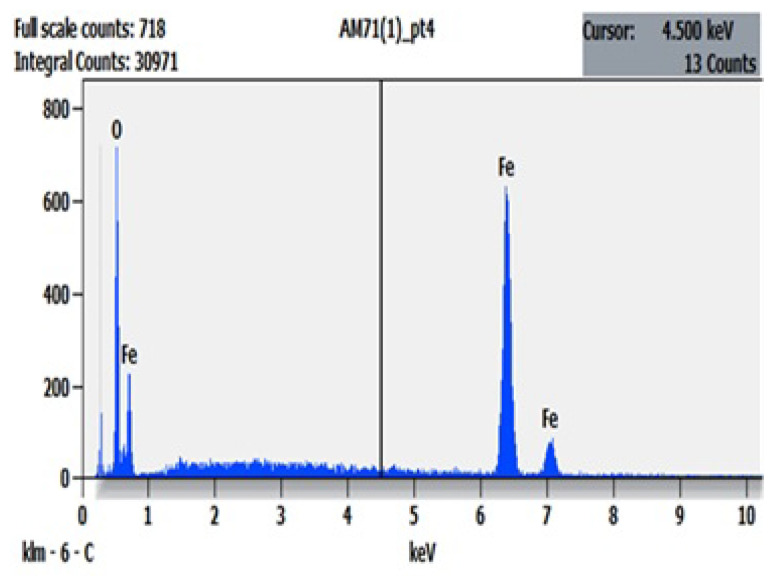
EDX spectral profile of the synthesized iron oxide nanoparticles.

EDX profiles were analyzed to identify the elemental composition of the samples. The EDX of Φ=1 (Figure 7) shows one peak for oxygen (O) (at ≈ 0.5 KeV) and three peaks for Fe (at ≈ 0.8, 6.4, and 7.1 keV), corresponding to their binding energies.

### 3.5. Analysis of the TEM images

**Figure 8:**
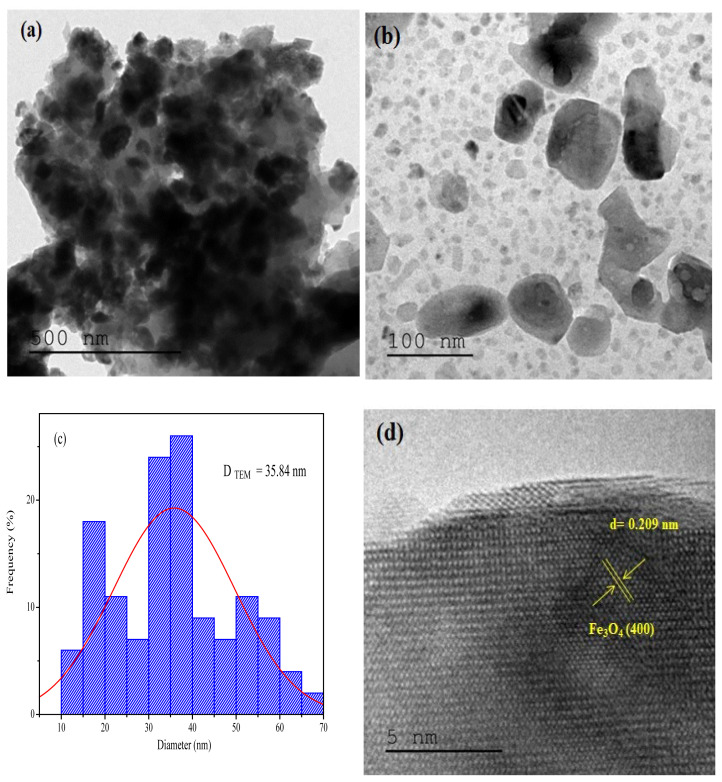
TEM images of magnetite nanoparticles (a,b), and size distribution histogram obtained from the TEM micrograph (c), and HRTEM micrograph with inter planar spacing of magnetite nanoparticles (d).

The Fe_3_O_4_ nanostructures (Φ = 1) were also characterized by TEM analysis (Figures 8a and 8b). The images reveal a high degree of dispersion achieved using the sample dispersion method. TEM images of the synthesized NPs crystals reveal the presence of spherical particles of uniform size. Well-crystallized morphology reliant on the applied fuel ratios was observed. The size distribution histogram obtained from the TEM measurements is presented in Figure 8c. The average particle size of the synthesized MNPs at Φ = 1 is approximately 35.84 nm. This is a narrow size distribution. The particle size of Fe_3_O_4_, obtained by analyzing the TEM images, agrees well with the particle size obtained by analyzing the XRD profiles. The interplanar spacing (d), determined from the HRTEM images, indicate the (400) lattice plane (Figure 8d).

### 3.6. Analysis of the TG profiles

**Figure 9:**
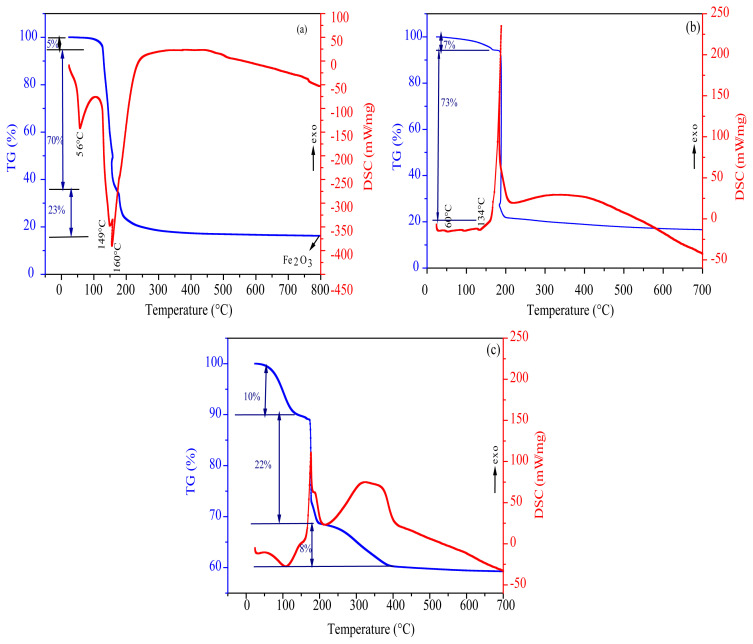
TG and DSC curves of Fe (NO_3_)_3_.9H_2_O (a), and the dried gels at different starch to ferric nitrate ratios Φ = 0.3 (b) and Φ = 1.7 (c).

The TG/DSC profiles of the Fe (NO_3_)_3_.9H_2_O precursor is presented in Figure 9a. The first two endothermic peaks at 56 °C and 149 °C, with mass loss 75%, indicate the removal of water of crystallization and nitrogen oxides.

A third endothermic peak appears in the temperature range of 149–225 °C. The DSC curve presents an intense slope. Thermal decomposition occurs under conditions of such high temperatures (mass loss: 23%). This is accompanied by the elimination of nitrogen, NO_2_, and water in the form of nitric acid (HNO_3_) and iron oxide (Fe_2_O_3_) (Figure 9a). The mode of decomposition of ferric nitrate is represented by Equations 6 and 7 as follows [56].


(6) $$\text{Fe }{\left({\text{NO}}_{3}\right)}_{3}.9{\text{H}}_{2}\text{O}\to \text{Fe }{\left({\text{NO}}_{3}\right)}_{3}+9{\text{H}}_{2}\text{O}{\;}_{\left(\text{g}\right)}$$

(7) $$\text{4}[\text{Fe }{\left({\text{NO}}_{3}\right)}_{3}]\to 2{\text{Fe}}_{2}{\text{O}}_{3\;(\text{s})}+12{\text{NO}}_{2\;(\text{g})}+3{\text{O}}_{2(\text{g})}$$

The combustion reactions occurring during the synthesis of iron oxide nanoparticles at fuel-lean conditions (Φ = 0.3) and fuel-rich conditions (Φ = 1.7) were analyzed using the TG-DSC technique. The temperature was raised to 700 °C from room temperature at a heating rate of 10 °C min^−1^ under an atmosphere of nitrogen (Figures 9b and 9c). The first stage (60 °C to 150 °C) of Figure 9b reveals the presence of two weak endothermic peaks that appear at 60 °C and 134 °C. The process occurring at this stage was accompanied by a weight loss of approximately 7%. Figure 9c reveals the presence of a broad endothermic peak in the region of 60–130 °C. The weight loss recorded at this stage was approximately 10%. The weight loss at this stage can be potentially attributed to the vaporization of residual water (from the precursor that was obtained after the drying process) and the decomposition of NH_4_NO_3_ (in the gelatinous mass) [30,31]. In the second temperature stage that ranges from 150 °C to 200 °C, a clear and sharp exothermic peak at approximately 200 °C was observed (Figure 9b). This stage was characterized by a high weight loss of 73% for dried gel under the lean fuel conditions (Φ = 0.3). The weight loss decreased to 22% at approximately 200 °C Figure 8c, when the starch to ferric nitrate ratio increased under rich conditions (Φ = 1.7). This temperature range (from 150 °C to 200 °C) is characteristic of the volatilization and the combustion reaction between ferric nitrate and starch in the gel with the release of H_2_O, CO_2_, and N_2_ gases [57]. In the third and last stage (above 200 °C), as shown in Figure 9b, no loss in weight was observed, indicating the decrease in rates of oxidation and decomposition of organic residues with the increase in temperature and starch to ferric nitrate ratio. Figure 9c reveals a high exothermic peak at the temperature range between 300 °C and 450 °C, accompanied by a weight loss of 8%.

### 3.7. Porosity characterization

**Figure 10:**
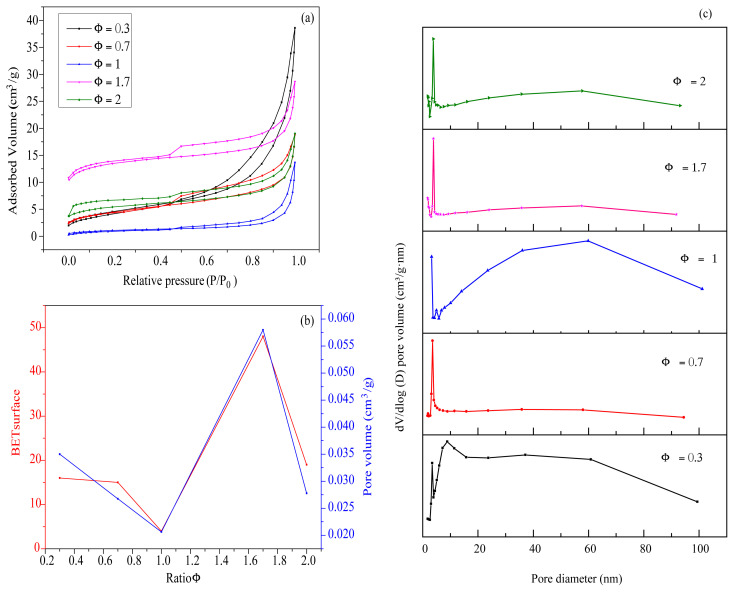
Nitrogen adsorption-desorption isotherms of the as-combusted powders at Φ = 0.3, Φ = 0.7, Φ = 1, Φ = 1.7, and Φ = 2 (a); and SBET and pore volume of iron oxide synthesized at different starch to ferric nitrate ratios (Φ) (b); and pore size distribution (c).

Figure 10a presents the N_2_ adsorption-desorption isotherms of iron oxides synthesized at different starch to ferric nitrate ratios (Φ = 0.3, 0.7, 1, 1.7, and 2). The recorded isotherms exhibited types II and III isotherm hysteresis loops, indicating mesoporous structures. As shown in Figure 10b, the specific surface areas (SSAs) and the pore volume of the powders depend on the starch to ferric nitrate ratio (Φ). The SSA of the products under conditions of varying Φ (Φ = 0.3, 0.7, 1, 1.7, and 2) were found to be 16, 15, 4, 48, and 19 m^2^ g^−1^, respectively. The pore volumes of the products at different Φ values were 0.035, 0.027, 0.020, 0.058 and 0.028 cm^3^ g^−1^, respectively. The maximum SSA (48 m^2^ g^−1^) was recorded at Φ = 1.7 (Figure 10b). This could be attributed to the rapid thermal decomposition process accompanied by the release of gases during the combustion reaction [57]. For Φ = 2, the decrease in the specific surface area can be attributed to the sintering process and the growth of the particle size between iron nitrate and starch [34]. The pore size diameter of the combusted powder at different starch to ferric nitrate ratios (Φ = 0.3, 0.7, 1, 1.7 and 2) were 14, 9, 37, 8, and 8 nm, respectively, indicating the mesoporous structure.

The BJH pore size distributions are illustrated in the inset of Figure 10c. The pore size distributions of the combusted powders at different starch to ferric nitrate ratios of Φ = 0.3, 0.7, 1, 1.7, and 2 were found to be 10, 4, 2.5, 3.5, and 4 nm, respectively. The pore size distribution at different ratios indicates a mesopore distribution.

### 3.8. UV-visible analysis

**Figure 11:**
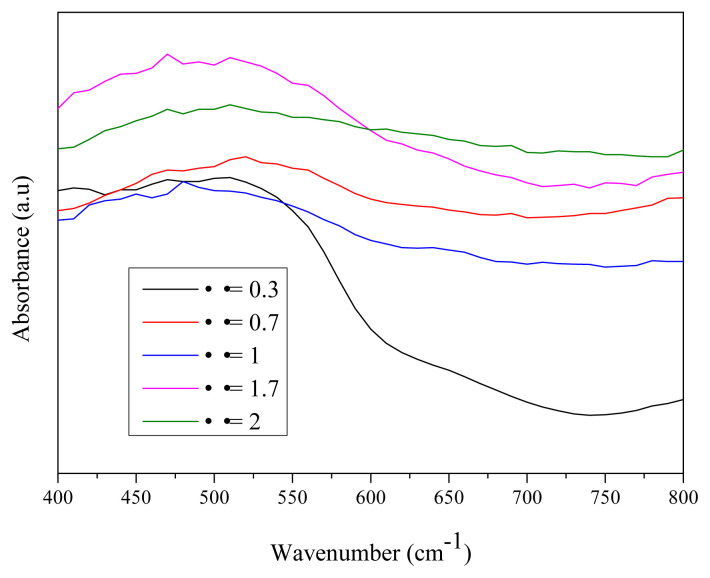
UV-vis DRS spectra of the resultant oxides at different starch to ferric nitrate ratios (Φ = 0.3, 0.7, 1, 1.7, and 2).

**Figure 12:**
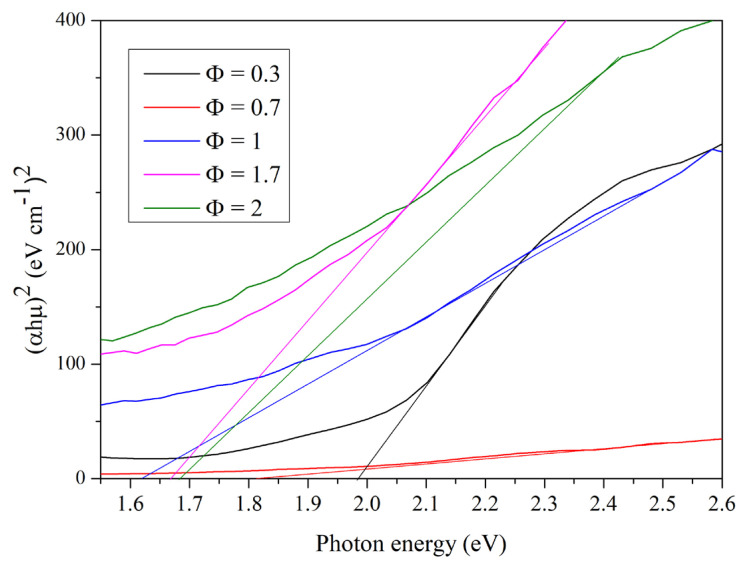
(αhμ)^2^ as function of photon energy hμ for determining the optical bandgap of iron oxide nanoparticles at different starch to ferric nitrate ratios (Φ = 0.3, 0.7, 1, 1.7, and 2).

The optical properties of the synthesized iron oxides at different starch to ferric nitrate ratios (Φ = 0.3, 0.7, 1, 1.7, and 2) were investigated by the UV-vis DRS spectroscopy. It can be seen from Figure 11 that the absorbance spectra of the iron oxide nanoparticles are remarkably different with the variation of starch content. As shown in the Figure 12, the curve of α- Fe_2_O_3_ (Φ = 0.3 and 0.7) has a strong photo absorption in the visible region, while the curve of Fe_3_O_4_ (Φ = 1, 1.7 and 2) was very wide. The optical energy band gap was calculated by the Tauc’s equation (Equation 8):


(8) $$a{\left(\alpha \text{h}\upsilon \right)}^{n}=\text{k }(\text{h}\upsilon -\text{Eg})$$

where α and k are absorption coefficient and optical transition-dependent constant of the material under investigation, hυ is the photon energy, and Eg is the band gap energy of the material. The exponent “n” indicates the nature of optical transition in the semiconductor. Both hematite and magnetite have a direct band gap (n = 2). A plot of (αhυ) ^2^ versus hυ is shown in Figure 12. The extrapolation of the linear portion of the (α.hυ) ^2^ versus the photon energy (hυ) axis provides the value of the optical band gaps Eg when (α.hυ) ^2^ is zero. The estimated optical band gaps energy of α-Fe_2_O_3_ at Φ = 0.3 and 0.7 are 1.99 and 1.82 which correspond with the reported value [58]. Table 2 presents the Band gap values of the prepared iron oxides at different starch to ferric nitrate ratios. This decrease of the value is due to the increase in the Φ ratio, the particle size increases that is responsible for decreasing the optical band gap [26]. The estimated optical band gaps energy of Fe_3_O_4_ at Φ = 1, 1.7 and 2 are 1.63, to 1.67 and 1.68, respectively (Table 2). The band gap energy increase with increasing of starch to ferric nitrate ratio. This increase of band gap energy is due to the decrease of the particle size of Fe_3_O_4_ nanoparticles [59]. From this observation, the difference in synthesized iron oxide nanoparticles can be attributed to the quantum size effect, with the size of the particles influencing their band gap energy [60].

### 3.9. Magnetic properties

**Figure 13:**
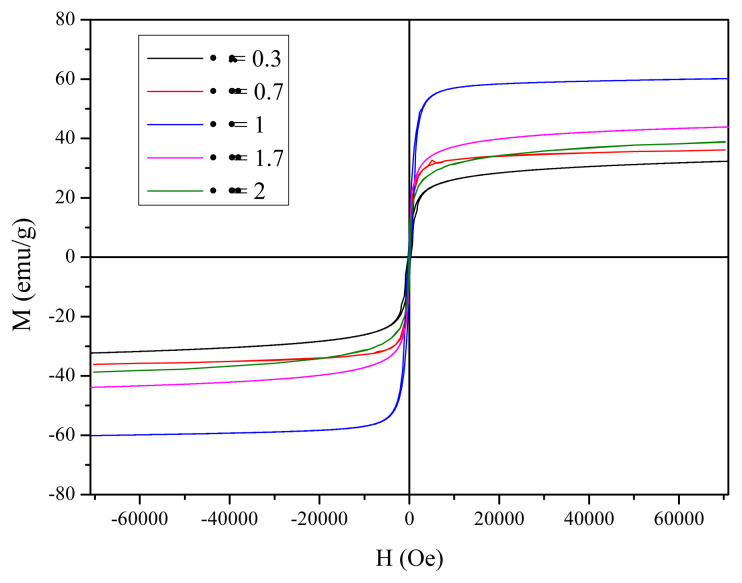
Magnetic hysteresis loop of the resultant iron oxide at different starch to ferric nitrate ratios Φ = 0.3, 0.7, 1, 1.7, and 2.

Figure 13 reveals the magnetic hysteresis loop of the resultant iron oxide at different starch to ferric nitrate ratios Φ = 0.3, 0.7, 1, 1.7, and 2 recorded at room temperature with the maximum field of 70 KOe. The saturation magnetization (M_s_), remanence (M_r_) and coercivity (H_c_) versus fuel ratio are listed in Table 3. It is remarkable that all the synthesized iron oxide nanoparticles are saturated at 70 KOe as well as the coercivity of the resultant iron oxide nanoparticles depend on the Φ ratios (Table 3). As shown in Figure 13, all the resultant iron oxide at different Φ ratios perform typical ferromagnetic properties at room temperature (Table 3). It is important to note that Φ has a substantial impact on the magnetic properties of the resultant iron oxides nanoparticles revealing that M_s_ decreases as Φ ratios increase [57]. Consequently, the synthesized α-Fe_2_O_3_ at Φ = 0.3 and 0.7 present the lowest saturation magnetization 32.15 and 36.19 emu/g (Table 3). It may be attributed to the largest particle size with the highest surface area and small crystallite size of hematite compared with those of the magnetite nanoparticle [31,34,61]. Obviously, the saturation magnetization gets the highest value (60.36 emu/g) at Φ = 1 which is due to the formation of ferromagnetic Fe_3_O_4_ phase with high crystallinity and large crystallite size (35.84 nm) as previously confirmed by XRD and FTIR analysis, whereas the saturation magnetization decreased to 38.95 emu/g at the highest Φ ratios (1.7 and 2). This may be attributed to the increase of the residual carbon in the Fe_3_O_4_ nanoparticles that led to the lowest crystallinity as exhibited by the XRD (Figures 4 and 5).

## 4. Conclusion

Homogenous hematite and magnetite crystalline phases have been synthesized following a new single fuel combustion method. In this method, ferric nitrate has been used as the oxidant and starch has been used as the reducing organic fuel (in different molar ratios). Starch has been effectively used in the low-temperature fuel approach. The thermal analysis of the starch-based precursor revealed that the final decomposition temperature, morphology, and the crystallization process of iron oxides are influenced by the starch ratio. Therefore, the maximum amount of the oxidant relative to the amount of fuel for Φ < 1 results in the formation of the maximum amounts of gases that form α-Fe_2_O_3_ as the main phase and Rietveld structure refinement analysis confirmed the formation of single Fe_3_O_4_ at Φ = 1. The α-Fe_2_O_3_ phase is reduced to Fe_3_O_4_ when the reducing gas (CO) is present under conditions of high fuel content (Φ ≥ 1). The porosity characterization indicated mesoporous structures of α-Fe_2_O_3_ and Fe_3_O_4_ phase with the highest specific surface area of 48 m^2^ g^−1^ at Φ = 1.7. The magnetic properties of Fe_3_O_4_ powders synthesized at Φ = 1 showed the highest saturation magnetization of 60.36 emu/g with high crystallinity. The proposed simple, fast, cheap, and environmentally friendly synthetic route can be considered as an alternative way to prepare pure hematite and magnetite in high quantity as the MNPs find their applications in various fields (e.g., biomedical field).

## Figures and Tables

**Table 1:** Required amount of starch and ferric nitrate and their corresponding Φ.

Molar ratio	Fe (NO_3_)_3_.9H_2_O (g; mol)	Starch (g; mol)	Φ
0.2	2; 0.005	0.169; 0.001	0.3
0.4	2; 0.005	0.339; 0.002	0.7
0.6	2; 0.005	0.508; 0.003	1
1	2; 0.005	0.847; 0.005	1.7
1.3	2; 0.005	1.017; 0.006	2

**Table 2:** Band gap of the resultant iron oxide at different starch to ferric nitrate ratios Φ.

Fuel ratio Φ	Band gap energy (eV)
**0.3**	1.98
**0.7**	1.82
**1**	1.63
**1.7**	1.67
**2**	1.68

**Table 3:** VSM magnetic parameters from major loop.

Fuel ratio Φ	Ms (emu/g)	Mr (emu/g)	Hc (Oe)
**0.3**	32.15	4.18	273.4
**0.7**	36.19	6.73	142.90
**1**	60.36	8.48	263.66
**1.7**	44.14	5.10	145.20
**2**	38.95	4.12	105.21
